# Single Cancer Center Experience on Patient Blood Management Eligibility in Oncological Surgery

**DOI:** 10.3390/jcm15072543

**Published:** 2026-03-26

**Authors:** Camilla L’Acqua, Roberto Lillini, Rosamaria Limuti, Flavio Arienti, Chiara Maura Ciniselli, Paolo Verderio, Ilaria Cavallo, Paolo Baili, Giulia Perrone

**Affiliations:** 1 Anesthesia and Intensive Care Unit, Fondazione IRCCS Istituto Nazionale dei Tumori, Via Giacomo Venezian, 1, 20133 Milano, Italy; camilla.lacqua@istitutotumori.mi.it (C.L.); rosamaria.limuti@istitutotumori.mi.it (R.L.); 2 Data Science Unit, Fondazione IRCCS Istituto Nazionale dei Tumori, Via Giacomo Venezian, 1, 20133 Milano, Italy; roberto.lillini@istitutotumori.mi.it (R.L.); lifetable@istitutotumori.mi.it (P.B.); 3 Immunohematology and Transfusion Medicine Service, Fondazione IRCCS Istituto Nazionale dei Tumori, Via Giacomo Venezian, 1, 20133 Milano, Italy; flavio.arienti@istitutotumori.mi.it; 4 Bioinformatics and Biostatistics Unit, Fondazione IRCCS Istituto Nazionale dei Tumori, Via Giacomo Venezian, 1, 20133 Milano, Italy; chiara.ciniselli@istitutotumori.mi.it (C.M.C.); paolo.verderio@istitutotumori.mi.it (P.V.); 5 Scientific Directorate, Fondazione IRCCS Istituto Nazionale dei Tumori, Via Giacomo Venezian, 1, 20133 Milano, Italy; ilaria.cavallo@istitutotumori.mi.it

**Keywords:** patient blood management, oncological surgery, anemia, transfusion risk, perioperative care

## Abstract

**Background:** Accurate identification of patients at high risk of perioperative blood transfusion is essential for optimizing patient blood management (PBM) strategies in oncological surgery. However, the performance of standard PBM eligibility criteria in real-world oncological settings remains incompletely characterized. **Material and Methods:** We conducted a retrospective, single-center analysis of 4228 consecutive patients undergoing elective oncological surgery of any complexity or liver transplantation over a 9-month period to assess transfusion need and estimate access to preoperative patient blood management (PBM) strategies to improve anemia management. Transfusion events were assessed within 24 h after surgery (PS24) and during the perioperative period (PO; 48 h before to 72 h after surgery). Two PBM eligibility strategies were applied to the same patient cohort and compared: (A) an observational approach, based on predefined PBM indicators (transfusion rate and transfusion index by surgical complexity), and (B) a multivariable modeling approach based on pre- and intraoperative anesthesiology assessment to estimate individual transfusion risk. Predictive performance of both strategies was evaluated using accuracy, Cramér’s V, area under the receiver-operating characteristic curve (AUC-ROC), and Brier score. **Results:** Overall, 7.7% of patients received transfusion within PS24 and 9.2% during PO. According to the observational approach, 23.8% of patients were classified as PBM-eligible, accounting for 89.2% of PS24 transfusions and 87.1% of PO transfusions. In the multivariable modeling approach, independent predictors of transfusion included surgical type (e.g., sarcoma surgery: OR 22.8 for PS24; OR 6.3 for PO; vs. senology surgery OR 1 for PS24; OR 1 for PO, respectively), anemia severity (moderate anemia: OR 64.3 and OR 107.9, respectively and mild anemia OR 3.38 and OR 3.65, respectively), high surgical complexity, operative time >3 h (>3 h: OR 8.83 and OR 8.65, respectively vs. <3 h OR 1 and OR 1, respectively), and ICU admission risk. The observational approach demonstrated stronger alignment with actual transfusion events (Cramér’s V = 0.44–0.47) and higher overall accuracy (90.8–92.3%); in contrast, a multivariable modeling approach showed superior discrimination (AUC = 0.94–0.95) and lower Brier scores, indicating better individual risk prediction. **Conclusions:** In a large real-world cohort of oncological surgical patients, standard PBM eligibility criteria effectively identified the majority of patients requiring perioperative transfusion. While multivariable modeling provided greater predictive precision, the observational PBM approach demonstrated strong clinical alignment and practical applicability. Integrating both strategies may support more effective transfusion risk stratification and PBM planning in oncological surgery.

## 1. Introduction

Preoperative anemia is a highly prevalent and clinically significant comorbidity, associated with adverse clinical outcomes, including increased morbidity and mortality, prolonged hospitalization, and impaired quality of life [1]. Although allogeneic blood transfusion remains a cornerstone in managing cancer-related anemia, accumulating evidence indicates that transfusion exposure constitutes an independent risk factor for reduced overall survival [2,3]. Multiple studies consistently demonstrated an association between perioperative transfusion and increased mortality, irrespective of cancer stage or therapeutic intent [4]. The adverse prognostic impact of transfusion is hypothesized to result from several mechanisms, including transfusion-related immunomodulation, increased susceptibility to infectious complications and other transfusion-associated adverse events. These findings emphasize the importance of implementing a restrictive and evidence-based transfusion approach, while reinforcing the clinical utility of patient blood management (PBM) programs in oncological care [5,6,7].

PBM is a multidisciplinary evidence-based framework to optimize patients’ blood resources. Its core pillars include identifying and treating preoperative anemia, minimizing perioperative blood loss, and enhancing patient’s physiological tolerance to anemia [6,8,9,10]. Robust evidence from multicenter observational cohorts and randomized controlled trials demonstrated that implementing PBM programs leads to improved clinical outcomes and decreased healthcare resource utilization, particularly in the perioperative period. Nonetheless, despite endorsement by the World Health Organization, the systematic integration of PBM into oncological surgical care remains limited and frequently underutilized [11].

This study reports the baseline assessment and methodological framework of a patient blood management (PBM) initiative implemented at the Fondazione IRCCS Istituto Nazionale dei Tumori (INT), Milan, Italy. The primary objective was to evaluate whether standardized criteria for preoperative PBM inclusion can reliably identify patients undergoing oncological surgery who are at an elevated risk of perioperative allogeneic blood transfusion. To assess transfusion risk, we employed and compared two approaches: an “observational approach” based on traditional PBM indicators, such as the transfusion rate and transfusion index, and a “multivariable modeling approach” incorporating patient-specific clinical characteristics and procedural complexity.

## 2. Materials and Methods

This retrospective, single-center study analyzed blood transfusion practices in elective oncological surgeries and liver transplant procedures performed between 26 September 2023 and 30 June 2024 at INT.

Clinical and administrative data were extracted from the institutional data warehouse (DWH), allowing comprehensive tracking of surgical pathways. Data sources included surgical scheduling systems, hospital admissions records, laboratory results, anesthetic evaluations and transfusion records.

Oncological surgeries were categorized by anatomical site and grouped into macro-categories according to their Surgical Complexity Score and bleeding risk, based on the 2018 classification proposed in the Position Paper of the Italian Society of Anesthesia and Intensive Care (SIAARTI) [12,13,14,15,16,17,18].

Anemia severity was defined according to the National Comprehensive Cancer Network (NCCN) classification (https://www.nccn.org/guidelines; accessed on 21 March 2026). Blood transfusions were evaluated within two distinct time frames: (a) early postoperative period: within 24 h after surgery (PS24), and (b) perioperative period: from 48 h before to 72 h after surgery (PO).

All analyses were performed on consecutive patients. The same cohort was evaluated using two different PBM eligibility strategies (observational vs. multivariable modeling approach). No theoretical or simulated groups and no before–after comparisons were performed.

Multivariable modeling was conducted using a complete-case (listwise deletion) approach. For anemia, cases with a missing anemia grade were excluded from the GLM. Similarly, observations with missing values for BMI, METS, dyspnea, and other covariates were excluded via listwise deletion.

(1) ***Approach A: Observational approach***

For each oncological surgery, the eligibility to PBM was based on the estimated surgical transfusion risk (STR) and presence of anemia (Hb < 130 g/L). Within each surgical specialty, a classification of surgical procedures was performed based on anatomical site, bleeding risk and surgical complexity score, as defined by the surgeon. In Table S1, for every specialty, a list of macro-categories is labeled (Supplementary Table S1).

To define the estimate STR, we evaluate the following key indicators of transfusion practices within each surgical macro-category and for each defined time frame (PS24 and PO):

- **Transfusion Rate (TR):** proportion of patients receiving at least one red blood cell (RBC) unit during the observation period, relative to the total number of patients undergoing the same surgical procedure. TR values were categorized as low (< 5%), intermediate (5–30%), or high (>30%) [19,20].
- **Transfusion Index (TI):** average number of RBC units transfused per patient, including both transfused and non-transfused patients. A TI value > 0.3 during the perioperative period (PO) was considered indicative of high blood consumption [19,20].

These indicators were calculated by surgical macro-category and for each defined time frame (Supplementary Table S1). TR within 24 h after surgery (TR-PS24) and TI during the perioperative period (TI-PO) were used to estimate the surgical transfusion risk (STR), which was classified as follows:

- **High-risk:** TR-PS24 > 30% in all patients.
- **Intermediate-risk:** TR-PS24 between 5 and 30% in all patients or TI-PO > 0.3 among anemic patients.
- **Low-risk:** TR-PS24 < 5% in all patients.

Based on this classification, eligibility for preoperative patient blood management (PBM) interventions was defined as follows:

- All patients undergoing high-risk surgeries.
- Patients with anemia of any grade (Hb < 130 g/L) undergoing intermediate-risk surgeries.
- Patients with moderate or severe anemia (Hb < 100 g/L) for low-risk surgeries.

(2) ***Approach B: Multivariable modeling approach***

All clinical variables collected from DWH were considered for analysis. The statistical workflow comprised the following steps: univariate analysis, bivariate comparisons; Chi-square (χ^2^) tests were applied to categorical variables, and Student’s *T*-tests were used for continuous variables.

To assess additional associations, univariate logistic regression analyses were performed for each dependent variable [21]. Subsequently, a backward stepwise generalized linear model (GLM) was constructed, employing a binomial distribution and a log link function to identify the strongest predictors of transfusion. For each covariate retained in the final model, odds ratios (ORs) with 95% confidence intervals were calculated [21]. Multicollinearity was assessed using variance inflation factors (VIFs). No significant collinearity was observed (all VIF values < predefined threshold) [21], supporting inclusion of the selected covariates in the final model (see Supplementary Table S2).

To assess whether the two groups of excluded cases for missing values in the variables and included cases differed systematically in their observed characteristics, we estimated a multivariable logistic regression model in which group membership was regressed on the full set of quantitative and categorical covariates. Unlike univariate comparisons (e.g., *T*-tests or chi-square tests), a multivariable approach allows the joint distribution of covariates to be considered simultaneously and accounts for potential confounding across characteristics [22,23]. This approach provides a more comprehensive assessment of between-group differences, as it evaluates whether groups differ conditionally on the entire set of observed covariates rather than on each characteristic separately. If the variables are not significantly associated with the groups, then the groups are comparable; if the variables are significantly associated with the group, then the groups are not comparable to each other via those variables and, therefore, are different.

In our case, we need to verify whether the groups are comparable to confirm that excluding missing cases does not alter the results.

(3) ***Comparison between approaches A and B***

Chi-square tests were used to assess statistical associations, and Cramér’s V was calculated to measure the strength of these associations [21].

Approach A reflects current PBM practice based on the transfusion rate and index derived from historical data, whereas approach B estimates the individual transfusion probability using multivariable regression.

Finally, to validate approach A, the accuracy score, Brier score [24] and AUC-ROC were also computed for the combined dataset of observed transfused patients and PBM eligibility [21].

The accuracy score reflects the proportion of correctly classified patients according to a predefined decision threshold and may be influenced by outcome prevalence. In contrast, the area under the receiver operating characteristic curve (AUC) measures discrimination, namely the ability of a model to correctly rank patients according to an increasing transfusion risk, independently of a specific cutoff. The Brier score evaluates the accuracy of probabilistic predictions by quantifying the mean squared difference between predicted probabilities and observed outcomes, thus incorporating elements of both discrimination and calibration. The accuracy score is calculated by dividing the number of correct predictions by the total prediction number. As you can see, accuracy can be easily described using the confusion matrix terms true positive, true negative, false positive, and false negative. Then, the Brier score computes the Yates, Sanders, and Murphy decompositions of the Brier mean probability score. The Brier score is a measure of disagreement between the observed outcome and a forecast (prediction).

Therefore, while accuracy is appropriate for evaluating binary eligibility strategies such as the observational PBM approach, the AUC and Brier score are more informative when assessing individualized probabilistic risk prediction models [21,24].

All the analyses were performed by considering a significance alpha level of 0.05 with Stata MP 17.0 software.

## 3. Results

During a 9-month observational period, 4228 consecutive patients undergoing oncological surgery or liver transplantation at INT were evaluated, included 49 pediatric patients (0–18 years old; 1.2% of all patients). For each patient, the type of surgery, estimated surgical transfusion risk (STR) and presence of preoperative anemia were assessed in relation to actual transfusion requirements (Table 1).

Transfusion events were categorized as no transfusion, transfusion PS24, and transfusion during PO. The mean age of the overall cohort was approximately 60 years. Regarding STR, 54.1% of all surgeries was categorized as low-risk, 37.4% as intermediate-risk, and 8.6% as high-risk. Surgical procedures associated with high STR (e.g., specifically colorectal, hepato-gastro-pancreatic, sarcoma surgeries) comprised a disproportionately large share of transfused cases (17–22%), despite accounting for a smaller portion of total surgeries. Preoperative anemia was a strong independent predictor of transfusion: approximately 75% of patients who received PO transfusions were anemic. Finally, 23.8% of patients met the criteria for inclusion in a PBM program based on an observational approach. Remarkably, this subgroup accounted for 87.1% of all PO transfusions.

Analysis of quantitative variables from the preoperative anesthesiology assessments revealed several statistically significant associations with transfusion requirements, as shown in Table 2.

Higher age was significantly associated (association checked by *T*-test) with PO transfusion (*p* = 0.022), while no statistically significant association was observed between age and PS24 (*p* = 0.290).

Statistically significant associations were observed between the majority of categorical clinical variables and transfusion requirements (see Table 3).

Sarcoma, gynecology and colorectal surgeries had the highest transfusion rates, compared to surgeries with lower complexity, such as senology. Also, anemia, the ASA score, a longer surgery duration (>3 h), and the surgical complexity score were strongly associated with transfusion as expected.

Multivariable logistic regression (approach B) identified several independent predictors significantly associated with transfusion requirements, both PS24 and PO (Table 4).

The final multivariable model included N = 3405 patients, corresponding to approximately 80.5% of the full cohort (N = 4228).

Anemia severity was the most powerful clinical predictor. Patients with moderate anemia had significantly higher odds of transfusion (OR = 64.3 [33.8–122.4] and OR = 107.9 [54.9–212.1]). Severe anemia was associated with extremely high odds of transfusion (OR = 61.3 [4.7–800.6] for PS24 transfusion). Among surgical variables, the type of surgery emerged as a strong determinant of transfusion risk. When comparing the types of surgery with a low risk of transfusion (i.e., senology), the highest odds were observed for sarcoma surgery (OR = 22.8 [95% CI: 9.2–56.5] for PS24 transfusion; OR = 6.3 [3.7–10.6] for PO transfusion), followed by gynecological surgery (OR = 17.1 [6.8–42.8] and OR = 4.9 [2.9–8.2]). Other strong predictors included a high surgical complexity score (OR = 10.3 [3.7–28.6] and OR = 6.2 [2.8–13.7] for high vs. low), duration of surgery > 3 h (OR = 8.8 [5.0–15.7] and OR = 8.6 [5.1–14.6]), and ICU admission risk (OR = 5.3 [3.7–7.7] and OR = 5.1 [3.6–7.3]). Taken together, these results validate the univariate analyses described above and confirm that anemia severity, surgical type and complexity, and surgical duration are the strongest independent predictors of transfusion in oncological surgery.

Missing values have no effect on the consistency of the results (Supplementary Table S3). In fact, there was no evidence of structural differences between the group of missing cases, originally excluded from the GLM model, and the included cases (almost all the characteristics presented *p* > 0.05).

Finally, a comparative analysis was conducted to evaluate the predictive performance of the approaches A and B in identifying patients requiring transfusion. The approach A identified 89.2% (290/325) of transfused patients in the PS24, as compared to 82.2% (267/325) by approach B. Contingency analysis (Table 5a) revealed a stronger association with the actual transfusion status for approach A (χ^2^ = 829.07, *p* < 0.001; Cramér’s V = 0.44) than for B (χ^2^ = 229.19, *p* < 0.001; Cramér’s V = 0.23). In terms of predictive metrics (Table 6a), approach A achieved higher overall accuracy (92.3%) with respect to B (87.5%), while B demonstrated better discrimination (AUC = 0.95 [95% CI: 0.94–0.96] vs. 0.85 [0.84–0.87]) and a lower Brier score (0.04 vs. 0.06).

Similar findings were observed for PO transfusions (Table 5b and Table 6b). Although approach B provided a superior predictive precision (higher AUC, lower Brier score), approach A demonstrated better alignment with actual transfusion events, particularly in terms of classification accuracy and association measures.

Given the low overall transfusion rate, additional classification metrics were calculated. For PS24 transfusions, the observational approach showed higher sensitivity (89.2%) compared to the multivariable model (49.9%), while specificity was comparable (81.6% vs. 79.9%). Both approaches demonstrated very high negative predictive values (>98% and almost 92%), reflecting the low event prevalence, whereas positive predictive values were modest (approximately 27 and 29%). Similar patterns were observed for PO transfusions (see Supplementary Table S4).

## 4. Discussion

Despite its single-center retrospective design, this study draws on a large, unselected patient cohort and high-quality integrated institutional data to generate real-world evidence on the performance of PBM eligibility strategies. The analysis was intended to assess the accuracy of different risk stratification approaches in identifying patients at increased risk of perioperative transfusion in oncology surgery.

The implementation of structured PBM programs has been consistently associated with a significant reduction in red blood cell transfusion requirements in patients undergoing oncological surgery [25]. As reduced transfusion exposure is linked to improved postoperative and long-term outcomes, an evidence-based and judicious approach to the identification and management of preoperative anemia remains essential [26,27].

Currently, PBM eligibility in major surgery is largely guided by preoperative hemoglobin levels and surgical complexity [28]. However, these criteria have not been specifically validated in oncological populations, where disease-related factors and procedural heterogeneity may substantially influence transfusion risk [29,30]. Moreover, given the time-sensitive nature of cancer surgery, preoperative anemia should not necessarily result in surgical delay; rather, it should prompt timely and targeted correction, through iron supplementation and optimization of erythropoiesis, as an integral component of routine perioperative care.

In this study, we compared two approaches to defining PBM eligibility: an observational model, based on preoperative hemoglobin levels and estimated transfusion risk; and a multivariable modeling approach incorporating detailed preoperative, anesthetic and surgical variables. Both models were evaluated across two timeframes (early postoperative period PS24 and perioperative period PO) to capture the variable clinical impact of transfusions throughout the surgical course.

Our findings demonstrated a strong and consistent association between the observational PBM eligibility criteria and actual perioperative transfusion risk in oncological surgery. High-risk surgical procedures and preoperative anemia emerged as independent and clinically meaningful predictors of transfusion. The disproportionately elevated transfusion rates observed in specific surgical categories, particularly sarcoma, gynecologic, and colorectal procedures, support a selective application of PBM interventions, enabling the focused allocation of resources to patients most likely to derive a benefit.

Overall, only 23.8% of patients were identified as PBM candidates according to the observational model; however, this subgroup accounted for nearly 90% of all transfusions, confirming the model’s strong discriminatory capacity. The multivariable analysis further validated the association between transfusion risk and several independent predictors, including surgical complexity, operative duration, ICU admission risk, and anemia severity—with particularly high odds ratios for moderate and severe anemia.

Comparative analysis revealed that, although the multivariable model achieved superior statistical discrimination, as reflected by higher AUC values, the observational approach demonstrated higher overall classification accuracy and stronger concordance with observed transfusion events (Cramér’s V = 0.44–0.47 vs. 0.23–0.25). These findings suggest that the observational PBM model represents a simple, pragmatic, and clinically reliable tool for identifying patients most likely to benefit from PBM interventions in complex oncological surgery.

While the observational PBM eligibility criteria captured the majority of transfused patients, approximately 11% of transfusion events occurred in patients not classified as PBM-eligible. This subgroup represents a clinically relevant “gray area” for which standard hemoglobin-based and procedural criteria may underestimate risk. To better characterized this gray zone population, a prospective validation within a structured PBM framework is warranted to reduce the possible confounding role of non-corrected preoperative anemia in restricted transfusion practice.

Additionally, to our knowledge, an international risk score is validated only for cardiological and traumatic surgery [31]. They identify about 25% of patients that could be classified as in the “gray area”, but we are not aware that such scores are also reliable in the oncological surgery setting.

Nevertheless, prospective validation within a structured PBM framework is warranted. For individual patients, a multidisciplinary tumor board approach may facilitate the development of personalized PBM strategies focused on the (1) optimization of preoperative hemoglobin levels; (2) integration of multimodal blood-sparing techniques; and (3) adoption of rational, evidence-based transfusion practices. A coordinated, patient-centered strategy is likely essential to improve perioperative outcomes and, ultimately, the long-term prognosis in surgical oncology patients [15].

This study has some limitations: a retrospective single-center design, missing data and incomplete clinical documentation.

Although the retrospective and single-center nature of this study may limit the external validity of the findings, the impact of this limitation appears mitigated by the large, consecutive, and unselected cohort analyzed. The consistency and magnitude of the observed associations across surgical categories and anemia strata, as well as the strong discriminatory performance of both PBM approaches, suggest that the main conclusions are robust and unlikely to be driven by center-specific artifacts.

Missing data were present for some clinical variables; however, their proportion was generally low and mainly involved secondary parameters (e.g., hemodynamic measures). Importantly, key predictors of the transfusion risk (anemia status, surgical type, surgical complexity, and operative time) were almost complete. Therefore, while missingness represents a methodological limitation, it is unlikely to have materially biased the main results or altered the observed risk stratification patterns.

Finally, incomplete documentation is an inherent limitation of real-world retrospective studies based on administrative and clinical databases. In this cohort, however, the availability of structured data from integrated institutional systems allowed for reliable capture of the main variables driving the transfusion risk. Given the very strong effect sizes observed in the multivariable models (e.g., for anemia severity and surgical complexity), residual information bias due to incomplete documentation is unlikely to substantially affect this study’s conclusions.

## 5. Conclusions

This single-center retrospective study evaluated two distinct strategies for identifying oncological surgical patients at increased risk of perioperative blood transfusion, applying both approaches to the same real-world cohort. Standard PBM eligibility criteria, based on the preoperative anemia status and procedure-specific transfusion risk, successfully identified nearly 90% of transfused patients.

Although a multivariable model incorporating detailed clinical and surgical variables achieved superior statistical discrimination, the observational PBM approach showed stronger alignment with actual transfusion practices and a higher classification accuracy. These findings suggest that routinely available PBM indicators provide a pragmatic and clinically meaningful tool for transfusion risk identification in complex oncological surgery, while the multivariable model primarily represents a benchmarking comparator for the observational PBM approach in order to make a future decision support tool.

Given the retrospective design, causal inferences regarding the effectiveness of PBM interventions cannot be drawn. Nevertheless, the results support the use of structured PBM eligibility criteria as a foundation for transfusion risk stratification and resource prioritization.

As practical guidance, PBM eligibility could be determined preoperatively through automated Electronic Medical Report (EMR)-based screening, which is not only feasible but also increasingly recommended as a part of modern perioperative optimization pathways. The implementation of preoperative anemia clinics as a core of PBM strategy and automated EMR flags can be used to identify PBM eligible patients preoperatively with digital perioperative pathways. Clinical resources could include a dedicated PBM team, standardized protocol for anemia work-up, clinical and informatics infrastructure, and training for clinicians and nurses to understand the alerts [32].

Prospective studies are warranted to evaluate how integrating observational and model-based approaches may improve PBM implementation and patient outcomes in oncological surgical care.

## Figures and Tables

**Table 1:** Patient characteristics.

	No Transfusion		Transfusion: PS24		Transfusion: PO		Totals	
	*Avg.*	*Std. Dev.*	*Avg.*	*Std. Dev.*	*Avg.*	*Std. Dev.*	*Avg.*	*Std. Dev.*
Age	60.2	15.7	61.3	15.1	62.1	15.4	60.4	15.7
	***N***	***%***	***N***	***%***	***N***	***%***	***N***	***%***
**Gender**								
M	1376	35.8	142	43.7	163	42.0	1539	36.4
F	2464	64.2	183	56.3	225	58.0	2689	63.6
**Anemia**								
No anemia	2637	68.7	79	24.3	97	25.0	2734	64.7
Yes anemia	1203	31.3	246	75.7	291	75.0	1494	35.3
**Type of Surgery**								
Colon–rectum surgery	221	5.8	56	17.2	70	18.0	291	6.9
Hepato-gastro-pancreatic surgery	254	6.6	60	18.5	69	17.8	323	7.6
Melanoma surgery	411	10.7	0	0.00	2	0.5	413	9.8
Eye surgery	132	3.4	0	0.00	0	0.0	132	3.1
Plastic and reconstructive surgery	438	11.4	5	1.5	9	2.3	447	10.6
Sarcoma surgery	201	5.2	73	22.5	78	20.1	279	6.6
Thoracic surgery	315	8.2	37	11.4	44	11.3	359	8.5
Gynecology	379	9.9	50	15.4	61	15.7	440	10.4
Otorhinolaryngology/maxillofacial surgery	264	6.9	8	2.5	15	3.9	279	6.6
Pediatrics	50	1.3	5	1.5	6	1.6	56	1.3
Senology	752	19.6	1	0.3	2	0.5	754	17.8
Urology	423	11.0	30	9.2	32	8.3	455	10.8
**Surgical transfusion risk (STR)**								
High-Risk	189	4.9	160	49.2	174	44.9	363	8.6
Intermediate-Risk	1384	36.0	154	47.4	195	50.3	1579	37.4
Low-Risk	2267	59.0	11	3.4	19	4.9	2286	54.1
**Preoperative PBM eligibility**								
No	3170	82.6	35	10.8	50	12.9	3220	76.2
Yes	670	17.4	290	89.2	338	87.1	1008	23.8
***Totals***	***3840***	***90.8***	***325***	***7.7***	***388***	***9.2***	***4228***	***100.0***

PS24: Early postoperative period: within 24 h after surgery. PO: Perioperative period: from 48 h before to 72 h after surgery.

**Table 2:** Association between observed transfusions and patients’ characteristics (continuous variables).

						Dependent Variable: *Transfusion: PS24*		Dependent Variable: *Transfusion: PO*	
Variable	Mean	Min.	Max.	SD	Missing (N)	*T*-Test	*p*-Value	*T*-Test	*p* (*p* < 0.05)
Age	60.37	2.86	101.16	15.66	0	−1.06	0.290	−2.29	0.022
Days between anesthesiology visit and surgery	18.71	0.00	120.00	18.34	60	3.69	<0.001	4.42	<0.001
Weight	69.37	13.00	144.00	15.59	68	2.74	0.006	3.11	0.002
Max. arterial pressure	133.03	78.00	207.00	20.58	250	2.97	0.003	3.27	0.001
Min. arterial pressure	83.75	50.00	802.00	15.46	250	3.37	0.001	3.87	<0.001
Heart rate (HR)	75.15	6.00	140.00	12.63	259	−5.75	<0.001	−5.68	<0.001

PS24: Early postoperative period: within 24 h after surgery. PO: Perioperative period: from 48 h before to 72 h after surgery.

**Table 3:** Association rates by odds ratios (OR) between observed transfusions and patients’ characteristics (bivariate logistic regression models between transfusion as dependent variable and the descriptive categorical variables as covariates).

			Dependent Variable: *Transfusion: PS24*		Dependent Variable: *Transfusion: PO*	
	Freq.	Percent	OR	*p*	OR	*p*
**Gender**						
F	2689	63.6	1 (ref.)	0.005	0.77	0.017
M	1539	36.4	0.72		1 (ref.)	
**Body mass index (BMI)**						
Underweight	139	3.3	1.58	0.006	1.48	0.005
Normal–Overweight	3462	81.9	1 (ref.)		1 (ref.)	
Obese	559	13.2	0.59		0.60	
*Missing*	*68*	*1.6*				
**Metabolic equivalent level (METS)**						
<4	187	4.4	1 (ref.)	0.015	1 (ref.)	0.000
>4	3964	93.8	0.55		0.41	
*Missing*	*77*	*1.8*				
**Dyspnea**						
No	3091	73.1	1 (ref.)	0.011	1 (ref.)	0.003
Heavy exertion	586	13.9	1.39		1.25	
Moderate exertion	411	9.7	1.66		1.80	
At rest	12	0.3	2.85		2.32	
*Missing*	*128*	*3.0*				
**Grade of anemia**						
No anemia	2390	56.5	1 (ref.)	0.000	1 (ref.)	0.000
Severe anemia	7	0.2	208.03		1 (empty)	
Moderate anemia	150	3.6	53.48		64.45	
Mild anemia	1337	31.6	4.35		4.44	
Anemia not known	344	8.1	1.25		1.39	
**Anemia**						
No anemia	2734	64.7	1 (ref.)	0.000	1 (ref.)	0.000
Yes anemia	1494	35.3	6.62		6.58	
**Anesthetic risk (ASA)**						
I	*279*	*6.6*	1 (ref.)	0.000	1 (ref.)	0.000
II	*2889*	*68.3*	6.40		7.55	
III	*979*	*23.2*	11.41		14.97	
IV	*21*	*0.5*	69.00		83.64	
*Missing*	*60*	*1.4*				
**ICU admission risk**						
No	3622	85.7	1 (ref.)	0.000	1 (ref.)	0.000
Yes	456	10.8	13.47		12.14	
*Missing*	*150*	*3.5*				
**Surgical category**						
Election	4195	99.2	1 (ref.)	0.000	1 (ref.)	0.000
Transplant	33	0.8	15.20		14.07	
**Surgical complexity score**						
Low	1729	40.9	1 (ref.)	0.000	1 (ref.)	0.000
Medium	1843	43.6	13.74		10.29	
High	590	14.0	49.05		36.90	
Not applicable	1	0.0	1 (empty)		1 (empty)	
*Missing*	*65*	*1.5*				
**Type of surgery**						
Colon–rectum surgery	291	6.9	179.44	0.000	119.10	0.000
Hepato-gastro-pancreatic surgery	*323*	*7.6*	171.79		102.14	
Melanoma surgery	*413*	*9.8*	1 (empty)		1.83	
Eye surgery	*132*	*3.1*	1 (empty)		1 (empty)	
Plastic and reconstructive surgery	*447*	*10.6*	8.52		7.73	
Sarcoma surgery	*279*	*6.6*	266.84		145.91	
Thoracic surgery	*359*	*8.5*	86.52		52.52	
Gynecology	*440*	*10.4*	96.54		60.52	
Otorhinolaryngology/maxillofacial surgery	*279*	*6.6*	22.23		21.36	
Pediatrics	*56*	*1.3*	73.82		45.12	
Senology	*754*	*17.8*	1 (ref.)		1 (ref.)	
Urology	*455*	*10.8*	53.15		28.44	
**Duration of surgery**						
Up to 3 h	2683	63.5	1 (ref.)	0.000	1 (ref.)	0.000
Over 3 h	1545	36.5	23.51		17.65	

PS24: Early postoperative period: within 24 h after surgery. PO: Perioperative period: from 48 h before to 72 h after surgery. ICU: Intensive care unit.

**Table 4:** Model approach: Final backward generalized linear model.

	Dependent Variable: *Transfusion: PS24*				Dependent Variable: *Transfusion: PO*			
Covariates	Odds Ratio	*p*	95% Lower CI	95% Upper CI	Odds Ratio	*p*	95% Lower CI	95% Upper CI
***Gender***								
M	1 (ref.)				1 (ref.)			
F	0.56	0.011	0.36	0.88	0.63	0.035	0.41	0.97
***Grade of anemia***								
No anemia	1 (ref.)				1 (ref.)			
Severe anemia	61.28	0.002	4.69	800.56				
Moderate anemia	64.30	<0.001	33.78	122.38	107.90	<0.001	54.89	212.11
Mild anemia	3.38	<0.001	2.34	4.89	3.65	<0.001	2.57	5.19
***ICU admission risk***								
No	1 (ref.)				1 (ref.)			
Yes	5.33	<0.001	3.69	7.70	5.09	<0.001	3.55	7.29
***Surgical complexity score***								
Low	1 (ref.)				1 (ref.)			
Medium	5.77	<0.001	2.21	15.09	3.64	<0.001	1.77	7.47
High	10.31	<0.001	3.71	28.65	6.23	<0.001	2.83	13.71
***Type of surgery***								
Senology	1 (ref.)				1 (ref.)			
Colon-rectal surgery	7.71	<0.001	3.24	18.35	2.48	<0.001	1.57	3.92
Hepato-gastro-pancreatic surgery	3.13	0.013	1.27	7.71	-			
Plastic and reconstructive surgery	7.84	0.003	2.06	29.80	4.08	0.001	1.74	9.58
Sarcoma surgery	22.83	<0.001	9.22	56.51	6.29	<0.001	3.74	10.58
Thoracic surgery	3.92	0.004	1.53	10.02				
Gynecology	17.10	<0.001	6.84	42.81	4.89	<0.001	2.91	8.21
Urology	4.32	0.002	1.71	10.88				
***Duration of surgery***								
Up to 3 h	1 (ref.)				1 (ref.)			
Over 3 h	8.83	<0.001	4.96	15.72	8.65	<0.001	5.14	14.58
**Weight**	0.98	<0.001	0.96	0.99	0.98	<0.001	0.97	0.99
**Heart Rate (HR)**	1.02	0.027	1.00	1.03	1.02	0.014	1.00	1.03
**Age**					1.02	0.001	1.01	1.03
*Constant*	*0.00*	*<0.001*	*0.00*	*0.00*	*0.00*	*<0.001*	*0.00*	*0.00*

PS24: Early postoperative period: within 24 h after surgery. PO: Perioperative period: from 48 h before to 72 h after surgery. ICU: Intensive care unit.

**Table 5:** Comparison between observational and model approaches in preoperative PBM eligibility.

(a) Transfusion: PS24.				
	**Predicted by observational approach**		**Predicted by model approach**	
**Preoperative PBM eligibility**	**No**	**Yes**	**No**	**Yes**
No	3185	35	2952	268
Yes	718	290	741	267
***Total***	***3903***	***325***	***3693***	***535***
Pearson chi2(1)	829.07	*p* < 0.001	229.19	*p* < 0.001
Cramér’s V	0.44		0.23	
(b) Transfusion: PO.				
	**Predicted by observational approach**		**Predicted by model approach**	
**Preoperative PBM eligibility**	**No**	**Yes**	**No**	**Yes**
No	3170	50	2947	273
Yes	670	338	717	291
***Total***	***3840***	***388***	***3664***	***564***
Pearson chi2(1)	941.93	*p* < 0.001	276.117	*p* < 0.001
Cramér’s V	0.47		0.26	

PS24: Early postoperative period: within 24 h after surgery. PO: Perioperative period: from 48 h before to 72 h after surgery. PBM: Patient blood management.

**Table 6:** Evaluation parameters of the comparison between observational and model approaches.

(a) Transfusion PS24.					
	**Accuracy**	**Brier Score**	**ROC Curve Area**	**95% Lower CI**	**95% Upper CI**
**Observational approach**	92.3%	0.06	0.85	0.84	0.87
**Model approach**	87.5%	0.04	0.95	0.94	0.96
(b) Transfusion: PO.					
	**Accuracy**	**Brier Score**	**ROC Curve Area**	**95% Lower CI**	**95% Upper CI**
**Observational approach**	90.8%	0.06	0.85	0.83	0.87
**Model approach**	86.6%	0.04	0.94	0.92	0.95

PS24: Early postoperative period: within 24 h after surgery. PO: Perioperative period: from 48 h before to 72 h after surgery.

## Data Availability

The datasets presented in this article are not readily available because of privacy and ethical restrictions. Requests to access the datasets should be directed to Giulia Perrone: giulia.perrone@istitutotumori.mi.it.

## Supplementary Materials

The following supporting information can be downloaded at https://www.mdpi.com/article/10.3390/jcm15072543/s1, Table S1: Classification of surgical complexity based on transfusion rate (TR) at INT at PS24 and transfusion index (TI) in the PO. Table S2: GLM covariates collinearity check by VIF. Table S3: Logistic multivariable analyses to test the comparability between cases excluded and included in the multivariable GLM model. Table S4: Scores of Sensitivity/Specificity for both approaches.

## Abbreviations

The following abbreviations are used in this manuscript:

PBM	Patient blood management
PS24	Transfusion events within 24 h after surgery
PO	Perioperative transfusion events
OR	Odds ratio
INT	Fondazione IRCCS Istituto Nazionale dei Tumori
DWH	Data WareHouse
SIAARTI	Italian Society of Anesthesia and Intensive Care
NCCN	National Comprehensive Cancer Network
TR	Transfusion rate
TI	Transfusion index
STR	Surgical transfusion risk
