# Supplementary material for: Single Cancer Center Experience on Patient Blood Management Eligibility in Oncological Surgery
Source: J Clin Med. 2026 Mar 26;15(7):2543. doi: 10.3390/jcm15072543 (PMC13072742; doi:10.3390/jcm15072543)
Supplement: Supplementary file 1 [file jcm-15-02543-s001.zip › jcm-4175192-supplementary.pdf]

Supplementary Table S1 - Classification of surgical complexity based on transfusion rate (TR) at INT at PS24 and transfusion index (TI) in the PO.

Type of surgery	TR-PS24			TI-PO		
	Anemic patients	Non-anemic patients	Total patients	Anemic patients	Non-anemic patients	Total patients
**COLORECTAL SURGERY**	**26.5%**	**11.0%**	**19.2%**	**1.15**	**0.30**	**0,75**
CCR1-CRS+HIPEC	87.5%	61.1%	73.5%	5.38	1.56	3,35
CCR2-CRS	100.0%	16.7%	37.5%	1.50	0.17	0.50
CCR3-INTESTINAL RESECTION LPT	45.5%	20.0%	37.5%	1.64	0.40	1.25
CCR4-LPS INTESTINAL RESECTION	17.1%	2.8%	9.9%	0.64	0.11	0.37
CCR5-OTHER SURGERY (Ileostomy, Recanalization, Exploratory LPS, other)	14.3%	0.0%	8.8%	0.46	0.06	0.31
**HEPATO-GASTRO-PANCREATIC SURGERY**	**30.8%**	**10.0%**	**18.6%**	**1.21**	**0.24**	**0.64**
CDB1-PANCREAS / SPLEEN / BILIARY TRACT	40.9%	19.0%	30.2%	1.36	0.38	0.88
CDB2-LIVER (MAJOR RESECTION)	26.9%	11.4%	17.1%	1.19	0.25	0.60
CDB3-ESOPHAGUS AND STOMACH	45.5%	0.0%	18.5%	0.82	0.00	0.33
CDB4-LIVER (MINOR RESECTION) / INTESTINAL RESECTION / LYMPHADENECTOMY	30.0%	3.6%	12.9%	0.77	0.11	0.34
CDB5-OTHER SURGERY (LPS diagnostics, Incisional hernia repair, Cholecystectomy, Jejunostomy, Other)	3.0%	0.0%	1.5 %	0.24	0.00	0.12
TRANSPLANT	90.9%	36.4%	54.5%	5.45	0.95	2.45
**MELANOMA SURGERY**	**0.0%**	**0.0%**	**0.0%**	**0.01**	**0.01**	**0.01**
CMO1-ELECTROCHEMOTHERAPY	0.0%	0.0%	0.0%	0.00	0.00	0.00
CMO2-DISSECTION	0.0%	0.0%	0.0%	0.09	0.05	0.06
CMO3-LYMPH NODE REMOVAL	0.0%	0.0%	0.0%	0.00	0.00	0.00
CMO4-RADICALIZATION + SENTINEL LYMPH NODE BIOPSY	0.0%	0.0%	0.0%	0.00	0.00	0.00
CMO5-OTHER SURGERY	0.0%	0.0%	0.0%	0.00	0.00	0.00
**OCULAR SURGERY**	**0.0%**	**0.0%**	**0.0%**	**0.00**	**0.00**	**0.00**
OCUL1-BRACHYTHERAPY/CLIP PLACEMENT	0.0%	0.0%	0.0%	0.00	0.00	0.00
OCUL2-OTHER SURGERY (Removal or demolition of lesion, resection, other)	0.0%	0.0%	0.0%	0.00	0.00	0.00
OCUL3-RECONSTRUCTION WITH GRAFT	0.0%	0.0%	0.0%	0.00	0.00	0.00
OCUL4-EYE ENUCLEATION	0.0%	0.0%	0.0%	0.00	0.00	0.00
OCUL5-SCLERAL BUCKLING	0.0%	0.0%	0.0%	0.00	0.00	0.00
**PLASTIC AND RECONSTRUCTIVE SURGERY**	**2.0%**	**0.7%**	**1.1%**	**0.07**	**0.04**	**0.05**
OCR1-RECONSTRUCTION WITH FLAP	16.7%	5.9%	8.7%	0.75	0.38	0.48
OCR2-RECONSTRUCTION WITH GRAFTS	0.0%	0.0%	0.0%	0.00	0.00	0.00
OCR3-MASTOPEXY	0.0%	0.0%	0.0%	0.00	0.00	0.00
OCR4-RECONSTRUCTION WITH PROSTHESIS/EXPANDERS	1.6%	0.0%	0.5%	0.02	0.00	0.01
OCR5-LIPOFILLING/ OTHER PROCEDURE	0.0%	0.0%	0.0%	0.00	0.00	0.00

Type of surgery	TR-PS24			TI-PO		
	Anemic patients	Non-anemic patients	Total patients	Anemic patients	Non-anemic patients	Total patients
**SARCOMA SURGERY**	**46.8%**	**12.5%**	**26.2%**	**2.09**	**0.35**	**1.04**
CDS1-RETROPERITONEUM	77.3%	33.3%	57.5%	3.39	0.83	2,24
CDS2-ARTS, CHEST WALL, SACRECTOMY	20.9%	2.9%	8.1%	0.58	0.08	0,22
CDS3-LPS, SPLENOPANCREASECTOMY	46.2%	17.6%	30.0%	3.69	0.94	2,13
CDS4-LIMB PERFUSION	0.0%	75.0%	75.0%	0.00	1.00	1.00
CDS5-OTHER SURGERY (Dissection, Exploratory LPS, Soft tissue biopsy, Other)	27.3%	0.0%	17.6%	0.91	0.00	0.59
**THORACIC SURGERY**	**23.3%**	**3.0%**	**10.3%**	**0.87**	**0.10**	**0.38**
OCT1-PNEUMO, THYMECTOMY, ESOPHAGECTOMY	0.0%	16.7%	11.1%	0.00	0.17	0,11
OCT2-LOBECTOMY OPEN	30.2%	6.3%	16.8%	1.25	0.25	0,69
OCT3-VATS	12.5%	0.8%	4.0%	0.31	0.02	0,10
OCT4-PLEURECTOMY	66.7%	0.0%	28.6%	4.00	0.00	1.71
OCT5-OTHER SURGERY (Mediastinoscopy, Bronchoscopy, Wall Demolition, Other)	25.0%	0.0%	11.1%	0.50	0.00	0.22
**GYNECOLOGY**	**21.6%**	**2.5%**	**11.3%**	**0.81**	**0.11**	**0,43**
OCG1-DEBULKING	44.9%	13.0%	32.2%	1.74	0.50	1,24
OCG2-LYMPHADENECTOMY	0.0%	0.0%	0.0%	0.00	0.00	0,00
OCG3-HYSTEROANNESSIECTOMY LPT/LPS	8.8%	0.0%	3.8%	0.37	0.03	0,18
OCG4-VULVECTOMY	7.7%	0.0%	3.2%	0.31	0.00	0.13
OCG5-HYSTEROSCOPY	12.9%	0.0%	4.9%	0.23	0.00	0.09
**OTORHINOLARYNGOLOGY / MAXILLOFACIAL SURGERY**	**10.7%**	**0.0%**	**2.9%**	**0.48**	**0.00**	**0.13**
ORL1-DEMOLITION + FLAP	42.9%	0.0%	14.3%	1.71	0.07	0.62
ORL2-DEMOLITION + SLC (SINGLE/BILATERAL)	15.4%	0.0%	4.4%	0.62	0.00	0.18
ORL3-NECK WITHOUT SLC (T/P Thyroidectomy, T/P Laryngectomy, T/P Pharyngectomy)	3.8%	0.0%	1.1%	0.15	0.00	0.05
ORL4-HEAD WITHOUT SLC (Parotid, Tongue, Mandible, Exenteratio Orbitae)	14.3%	0.0%	4.1%	0.86	0.00	0.24
ORL5-SKIN/DIAGNOSIS/SMALL EXERTION (LASER CORDECTOMY)	0.0%	0.0%	0.0%	0.00	0.00	0.00
**PEDIATRICS**	**14.7%**	**0.0%**	**8.6%**	**0.44**	**0.00**	**0.26**
PED1-THORAX	30.0%	0.0%	21.4%	1.20	0.00	0.86
PED2-ABDOMINAL (Urology, Sarcomas, Gynecology, Colon & Rectum)	22.2%	0.0%	13.3%	0.33	0.00	0.20
PED3-ENT	0.0%	0.0%	0.0%	0.00	0.00	0.00
PED4-OTHER SURGERY	0.0%	0.0%	0.0%	0.00	0.00	0.00
**SENOLOGY**	**0.0%**	**0.2%**	**0.1%**	**0.00**	**0.01**	**0.00**
OCB1-MASTECTOMY + AXILLARY DISSECTION	0.0%	0.0%	0.0%	0.00	0.00	0.00
OCB2-MASTECTOMY	0.0%	0.8%	0.5%	0.00	0.01	0.01
OCB3-QUADRANTECTOMY + AXILLARY DISSECTION	0.0%	0.0%	0.0%	0.00	0.00	0.00

Type of surgery	TR-PS24			TI-PO		
	**Anemic patients**	**Non-anemic patients**	**Total patients**	**Anemic patients**	**Non-anemic patients**	**Total patients**
OCB4-QUADRANTECTOMY AND/OR SENTINELYMPH NODE BIOPSY	0.0%	0.0%	0.0%	0.00	0.01	0,01
OCB5-LIPOFILLING/OTHER SURGERY	0.0%	0.0%	0.0%	0.00	0.00	0,00
**UROLOGY**	**16.7%**	**2.5%**	**6.6%**	**0.51**	**0.10**	**0,21**
OCU1-PROSTATECTOMY	33.3%	0.0%	7.7%	0.67	0.00	0.15
OCU2-RPLND	23.1%	5.7%	9.1%	1.38	0.11	0.36
OCU3-NEPHRECTOMY/RENAL RESECTION/ADRENECTOMY	29.4%	5.4%	13.0%	0.59	0.22	0.33
OCU4-CYSTECTOMY	38.5%	11.1%	27.3%	1.46	1.00	1.27
OCU5-OTHER SURGERY (TURB, Cystoscopy, Ureteroscopy, Penile Amputation, Orchiectomy, Orchifuniculectomy, Inguinal-Pelvic lymphadenectomy, Other)	4.3%	0.0%	1.2%	0.12	0.00	0.03

PS24: Early postoperative period: within 24 hours after surgery.

PO: Perioperative period: from 48 hours before to 72 hours after surgery.

CRS: Citoreductive surgery, HIPEC: Hyperthermic intraperitoneal chemotherapy, LPS: Laparoscopy; LPT: Laparotomy; SLC: Laterocervical emptying; TURB: Transurethral resection of bladder; T/P: Total or Partial; VATS: Video-Assisted Thoracic Surgery, RPLND: Retroperitoneal Lymph node dissection.

Supplementary Table S2– GLM covariates collinearity check by VIF.

	Dependent variable: Transfusion: PS24	Dependent variable: Transfusion: PO
**Covariates**	**VIF**	**VIF**
Gender		
M	10.00	10.00
F	1.95	1.89
Grade of anemia		
No anemia	3.55	3.53
Severe anemia	1.02	
Moderate anemia	1.43	1.42
Mild anemia	3.41	3.38
ICU admission risk		
No	10.00	10.00
Yes	1.27	1.26
Surgical complexity score		
LOW	9.40	9.36
MEDIUM	9.55	9.52
HIGH	4.70	4.68
Type of surgery		
Senology	1.92	1.63
Colon-rectal surgery	1.50	1.20
Hepato-gastro-pancreatic surgery	1.49	
Plastic and reconstructive surgery	1.52	1.35
Sarcoma surgery	1.36	1.14
Thoracic surgery	1.47	
Gynecology	1.69	1.41
Urology	1.52	
Duration of surgery		
Up to 3 hours	10.00	10.00
Over 3 hours	1.84	1.76
Weight	1.36	1.36
Heart Rate (HR)	1.04	1.06
Age		1.07

PS24: Early postoperative period: within 24 hours after surgery.
PO: Perioperative period: from 48 hours before to 72 hours after surgery.
ICU: Intensive Care Unit.

Collinearity between covariates in GLMs was tested considering only the variables and their categories remained in the GLMs after internal collinearity check and backward selection: After dichotomization of every variable remained in the model the VIF of the covariates was computed and a common critical threshold of VIF ≤ 10.00 was considered to accept the variable.

Supplementary Table S3 – Logistic multivariable analyses to test the comparability between cases excluded and included in the multivariable GLM model.

	**Excluded cases**		**Dependent variable: *Included vs. Excluded***			
**Covariates**	**N**	**%)**	**Odds ratio**	**p**	**95% Lower CI**	**95% Upper CI**
Gender						
M	343	41.7	1 (ref.)			
F	480	58.3	0.95	0.765	0.67	1.34
Grade of anemia						
No anemia	502	61.0	1 (ref.)			
Severe anemia	7	0.9	1			
Moderate anemia	31	3.8	1.14	0.701	0.57	2.28
Mild anemia	229	27.8	1.08	0.586	0.82	1.42
Anemia not known	54	6.6				
ICU Admission risk						
No	581	70.6	1 (ref.)			
Yes	92	11.2	0.97	0.887	0.63	1.49
Missing	*150*	*18.2*				
Surgical complexity score						
Low	287	34.9	1 (ref.)			
Medium	354	43.0	1.24	0.218	0.88	1.75
High	116	14.1	0.78	0.336	0.46	1.30
Missing	*65*	*8.0*				
Type of surgery						
Senology	101	12.3	1 (ref.)			
Colon-rectal surgery	35	4.3	1.24	0.483	0.68	2.23
Hepato-gastro-pancreatic surgery	71	8.6	2.75	0.009	1.28	5.89
Melanoma surgery	93	11.3	0.86	0.523	0.53	1.38
Plastic and reconstructive surgery	52	6.3	1.90	0.019	1.11	3.25
Sarcoma surgery	54	6.6	0.85	0.559	0.49	1.48
Thoracic surgery	78	9.5	1.98	0.054	0.99	3.95
Gynecology	54	6.6	0.89	0.624	0.55	1.43
Otorhinolaryngology/maxillofacial surgery	63	7.7	0.57	0.027	0.34	0.94
Pediatrics	32	3.9	0.14	0.000	0.06	0.33
Urology	58	7.1	0.98	0.946	0.59	1.63
Duration of surgery						
Up to 3 hours	531	64.5	1 (ref.)			
Over 3 hours	292	35.5	0.97	0.847	0.68	1.36
	**Mean**	**Standard deviation**				
**Weight**	70.28	17.45	1.00	0.970	0.99	1.01
Missing	*68 cases*					
**Heart Rate (HR)**	74.74	13.45	1.00	0.489	0.99	1.01
Missing	*259 cases*					
**Age**	59.54	17.66	0.99	0.131	0.99	1.00
Missing	*0 cases*					
Constant			20.03	0.000	5.72	70.22

ICU: Intensive Care Unit.

Supplementary Table S4 – Scores of Sensitivity/Specificity for both approaches.

	PS24 - Observational approach	PS24 - Model approach
Sensitivity	89.2%	49.9%
Specificity	81.6%	79.9%
PPV	28.8%	26.5%
NPV	98.9%	91.7%
	**PO - Observational approach**	**PO - Model approach**
Sensitivity	87.1%	51.6%
Specificity	82.6%	80.4%
PPV	33.5%	28.9%
NPV	98.4%	91.5%

PPV = Positive Predictive Value.
NPV = Negative Predictive Value.
